# Supplementary material for: Identifying the role of NLRP3 inflammasome in stroke progression and outcome before recanalization
Source: Cell Rep Med. 2026 Mar 31;7(4):102723. doi: 10.1016/j.xcrm.2026.102723 (PMC13130646; doi:10.1016/j.xcrm.2026.102723)
Supplement: Document S1. Figures S1 and S2 and Tables S1–S3 [file mmc1.pdf]

Cell Reports Medicine, Volume *7*

Supplemental information

Identifying the role of NLRP3 inflammasome in stroke progression and outcome before recanalization

Maximilian Bellut, Alexander M. Kollikowski, Marius L. Vogt, Lukas Rossnagel, Ibrahim Hawwari, Bernardo S. Franklin, Mirko Pham, Guido Stoll, and Michael K. Schuhmann

Supplemental Figure 1 (related to Figure 1): Hyperacute inflammasome activation and neuroprotective effects of MCC950 during experimental cerebral ischemia

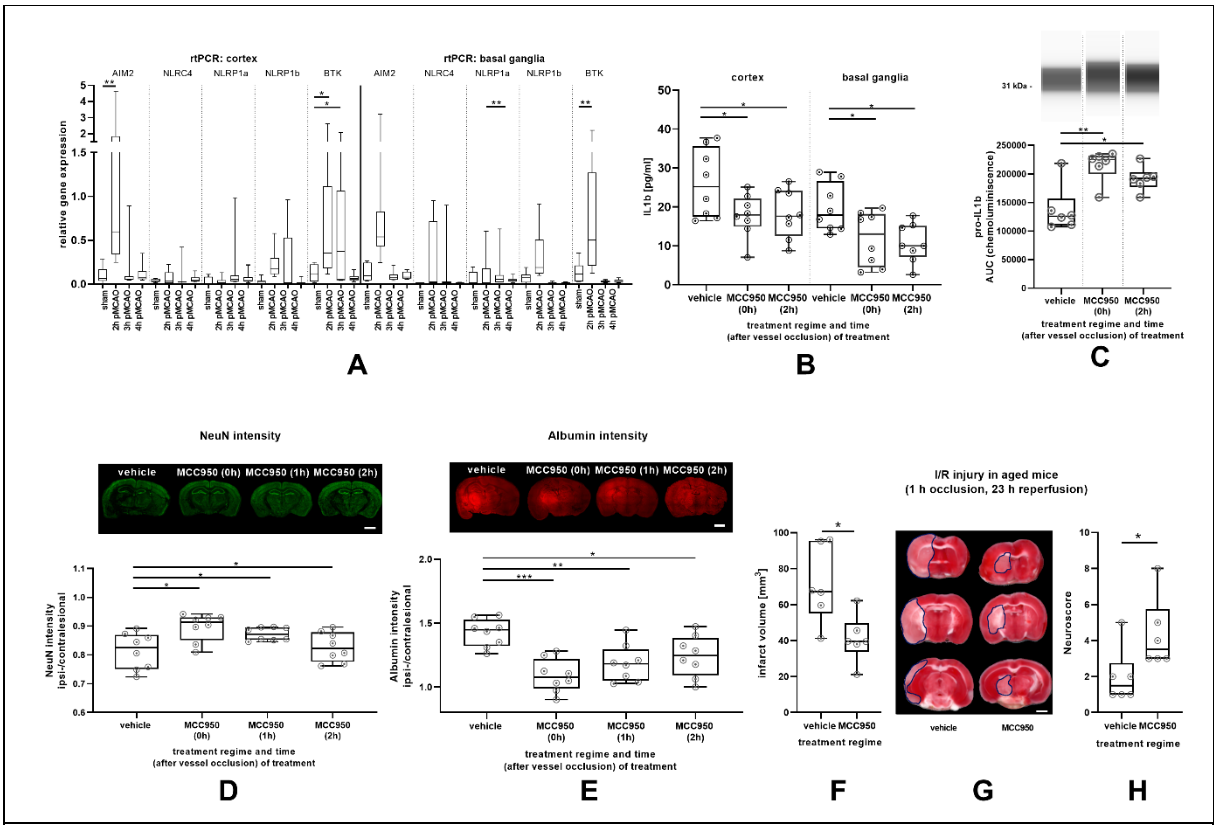

Suppl. Fig. 1 (A) Relative gene expression of AIM2, NLRC4, NLRP1a, NLRP1b and BTK in the ischemic cortices and basal ganglia of mice during permanent middle cerebral artery occlusion (pMCAO) for 2 h, 3 h and 4 h or sham-treatment; n = 8-10 per group. Data was analyzed by 1-way ANOVA. (B) Cytometric bead array to quantify IL1b protein levels of ipsilateral cortical and basal ganglial brain lysates after 4 h of MCAO of vehicle-, MCC950 (0h)- and MCC950 (2h)-treated mice; n = 8 per group. Data was analyzed by 1-way ANOVA. (C) WES capillary electrophoresis and immunoblotting of pro-IL1b in ipsilateral cortical brain lysates after 4 h of MCAO of vehicle-, MCC950 (0h)- and MCC950 (2h)-treated mice. Top: Representative immunoblotting results of 1 individual per group as assigned. Bottom: pro-IL1b levels measured by WES capillary electrophoresis quantified as area under the curve (AUC). Data was analyzed by unpaired t-test; n = 6 per group. (D) Top: Representative immunohistochemical stainings of NeuN (neurons, green) of coronary brain slices after 4 h of MCAO of vehicle-, MCC950 (0h), MCC950 (1h)- and MCC950 (2h)-treated mice using 5 × objective. Scale bar = 2 mm. Bottom: Ratio of ipsilesional to contralesional NeuN intensity of the aforementioned groups; n = 8 per group. Data was analyzed by 1-way ANOVA. (E) Top: Representative immunohistochemical stainings of Albumin (red) of coronary brain slices after 4h of MCAO of vehicle-, MCC950 (0h), MCC950 (1h)- and MCC950 (2h)-treated mice using 5 × objective. Scale bar = 2 mm. Bottom: Ratio of ipsilesional to contralesional Albumin intensity of the aforementioned groups; n = 8 per group. Data was analyzed by 1-way ANOVA. (F) Infarct size comparison in mm^3 of vehicle- and MCC950-treated mice euthanized after 1h of tMCAO and 23 h of reperfusion; n = 6 per group. (G) Representative TTC stainings of vehicle- and MCC950-treated mice euthanized after 1h of tMCAO and 23h of reperfusion to visualize infarct volumes. Infarcts circled by a blue line. Scale bar = 2 mm. (H) Neuroscore as clinical testing of the aforementioned treatment groups for their respective clinical outcomes; n = 6 per group. Data were analyzed by 1-way ANOVA. Results are presented as box plots indicating the median, 25th/75th percentile, minimum and maximum. *$p < 0.05$, **$p < 0.01$, ***$p < 0.001$.

Supplemental Figure 2 (related to Figure 3): Compartment-specific inflammasome signaling and immune cell dynamics in systemic versus ischemic human stroke blood

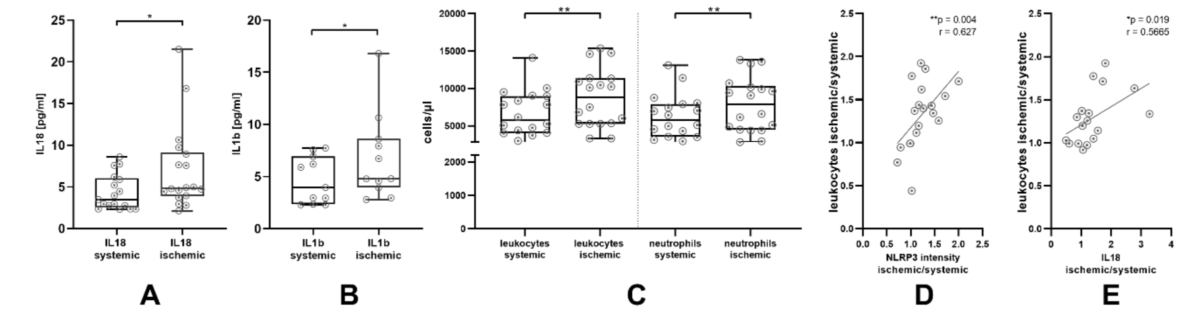

Suppl. Fig. 2. (A) Cytometric bead array to detect IL18 concentrations in the systemic and ischemic compartment from human blood samples. Data was analyzed by paired t-test; n = 18 (B) Cytometric bead array to detect IL1b concentrations in the systemic and ischemic compartment from human blood samples. Data was analyzed by paired t-test; n = 11. (C) Total leukocyte and neutrophil counts of systemic arterial blood versus local occlusive blood. Data was analyzed by unpaired t-test; n = 18. (D) Proportion of the ischemic/systemic leukocyte count ratio to the ischemic/systemic NLRP3 intensity ratio. Data was analyzed by Spearman correlation; n = 18. (E) Proportion of the ischemic/systemic leukocyte count ratio to the ischemic/systemic IL18 concentration ratio. Data was analyzed by Spearman correlation; n = 18. Results are presented as box plots indicating the median, $25^{th}/75^{th}$ percentile, minimum and maximum. *p < 0.05, **p < 0.01, ***p < 0.001.

**Baseline ASPECTS**						
**Predictor variable**	**B**	**SE**	**95% CI**	**t**	R^2	**p** value
NLRP3⁺ cells$_{ischemic}$	-0.097	0.035	-0.17 – 0.023	-2.80	0.33	0.013
Age	0.033	0.035	-0.042 – 0.11	0.93	0.051	0.37
Female sex	0.3	0.95	-1.71 – 2.31	0.32	0.0062	0.76
History of prior stroke	1.07	1.11	-1.27 – 3.42	0.97	0.055	0.35
Unknown onset	0.6	1.05	-1.62 – 2.82	0.57	0.02	0.57
Antiplatelet drugs	0.6	1.05	-1.62 – 2.82	0.57	0.02	0.57
Anticoagulants	1.15	1.02	-1.0009 – 3.31	1.14	0.074	0.27
**NIHSS after 72h**						
**Predictor variable**	**B**	**SE**	**95% CI**	**t**	R^2	**p** value
NLRP3⁺ cells$_{ischemic}$	0.71	0.14	0.4 – 1.022	4.9	0.63	0.0002
Age	0.042	0.2	-0.39 – 0.47	0.21	0.003	0.84
Female sex	-6.19	5.098	-17.12 – 4.74	-1.21	0.095	0.24
History of prior stroke	-8.26	6.45	-22.08 – 5.57	-1.28	0.1	0.22
Unknown onset	-3.16	5.67	-15.33 – 9.0059	-0.56	0.022	0.59
Onset-to-recanalization time	-0.0058	0.014	-0.037 – 0.025	-0.41	0.019	0.69
Antiplatelet drugs	-6.5	5.89	-19.13 – 6.13	-1.1	0.08	0.29
Anticoagulants	1.15	1.016	-1.0009 – 3.31	1.13	0.075	0.27
Intravenous alteplase treatment	7.71	4.95	-2.89 – 18.33	1.56	0.15	0.14
**mRS at discharge**						
**Predictor variable**	**B**	**SE**	**95% CI**	**t**	R^2	**p** value
NLRP3⁺ cells$_{ischemic}$	0.075	0.034	0.00039 – 0.15	2.13	0.22	0.049
Age	0.037	0.033	-0.032 – 0.11	1.14	0.076	0.27
Female sex	0.35	0.89	-1.53 – 2.23	0.39	0.0096	0.69
History of prior stroke	-0.89	1.041	-3.1 – 1.31	-0.86	0.044	0.4
Unknown onset	-0.89	0.96	-2.93 – 1.15	-0.93	0.051	0.37
Onset-to-recanalization time	0.0019	0.0017	-0,0018 - 0.0057	1.13	0.1	0.28
Antiplatelet drugs	0.49	0.98	-1.59 – 2.57	0.5	0.016	0.62
Anticoagulants	-0.62	0.98	-2,69 – 1.45	-0.63	0.024	0.54
Intravenous alteplase treatment	1.33	0.82	-0.41 – 3.072	1.63	0.14	0.12
**mRS after 3 months**						
**Predictor variable**	**B**	**SE**	**95% CI**	**t**	R^2	**p** value
NLRP3⁺ cells$_{ischemic}$	0.12	0.055	0.0023 – 0.24	2.27	0.34	0.047
Age	0.011	0.047	-0.094 – 0.12	0.23	0.0052	0.82
Female sex	-0.86	1.35	-3.86 – 2.14	-0.64	0.039	0.54
History of prior stroke	0.6	1.81	-3.43 – 4.63	0.33	0.011	0.75
Unknown onset	-0.89	0.96	-2.93 – 1.15	-0.93	0.051	0.368
Onset-to-recanalization time	0.0061	0.0068	-0.0096 – 0.022	0.89	0.091	0.39
Antiplatelet drugs	-0.22	1.56	-3.7 – 3.26	-0.14	0.002	0.89
Anticoagulants	0.38	1.43	-2.81 – 3.56	0.26	0.0068	0.79
Intravenous alteplase treatment	0.86	1.35	-2.14 – 3.86	0.64	0.039	0.54

Suppl. Table 1: Exploratory univariate regression analysis of patient characteristics, NLRP3⁺ cells and their association with early infarct volume and outcome measures. ASPECTS, Alberta Stroke Program Early CT Score; B, Coefficient; CI, Confidence interval; mRS, modified Rankin Scale; NIHSS, National Institutes of Health Stroke Scale; R2, Coefficient of determination; SE, standard error.

**mRS after 3 months**						
**Predictor variable**	**B**	**SE**	**95% CI**	**t**	**R^2**	**p** value
Constant	-1.24	1.29	-4.4 – 1.91	-0.96		0.37
$NLRP3^+$ $cells_{ischemic}$	0.16	0.048	0.038 – 0.27	3.22	0.63	0.018

Suppl. Table 2: Best-fit model in prediction of long-term outcome at 3-month follow-up. Multivariable regression using stepwise-backwards selection (cut-off point of p > 0.1); Covariates: age, NIHSS (National Institutes of Health Stroke Scale), onset-to-recanalization time; R^2-adjusted = 0.57; multiple correlation coefficient = 0.79. B, Coefficient; CI, Confidence interval; R^2, Coefficient of determination; SE, standard error.

**Cytokines**	***IL18***	***IFNγ***	***IL10***	***IL8***	***IL1b***	***IL6***	***MCP1***	***IL12p70***	***IL23***	***IFNα2***	***IL17A***	***TNFα***	***IL33***
**Pat. no. below detection limit**	0	8	1	1	7	2	0	17	16	18	18	18	18
**mean conc. systemic**	4.35 (±2.12)	2.69 (±2.35)	9.04 (±8.25)	255.17 (±210.8)	4.65 (±2.23)	26.25 (±22.10)	285.14 (±149.6)	4.53 -	11.92 (±1.76)	< LoD	< LoD	< LoD	< LoD
**mean conc. ischemic**	7.05 (±2.09)	3.89 (±3.15)	10.76 (±8.91)	434.68 (±250.7)	6.78 (±4.14)	37.65 (±26.47)	294.98 (±155.1)	7.43 -	6.53 (±0.69)	< LoD	< LoD	< LoD	< LoD
**p-value cytokine conc. i/s**	***0.048***	0.051	0.453	0.156	***0.043***	***0.023***	0.811	-	-	-	-	-	-
**p-value correlation cytokine conc. [i/s]/ NLRP3 intensity [i/s]**	***0.0001***	0.711	0.128	0.413	*0.08*	0.217	0.685	-	-	-	-	-	-
**r^2 correlation cytokine conc. [i/s]/ NLRP3 intensity [i/s]**	0.88	0.02	0.14	0.04	0.39	0.11	0.11	-	-	-	-	-	-

Suppl. Table 3: Panel showing cytokine concentrations (IL18, IFNγ, IL10, IL8, IL1b, IL6, MCP1, IL12p70, IL23) in systemic and ischemic (pial) blood samples from stroke patients undergoing endovascular thrombectomy (n = 18). The panel was selected for IL1b and IL18 detection; several other analytes were below the limit of detection, reported as < LoD
For each cytokine, the following information is provided: number of patients with levels below the detection limit, mean concentration in systemic and ischemic blood samples, p-value comparing systemic vs. ischemic concentrations and the correlation (p-value and r^2) between the ischemic/systemic cytokine ratio and the corresponding ischemic/systemic NLRP3 intensity ratio. i/s = ischemic/systemic.
