# Supplementary material for: Identifying the role of NLRP3 inflammasome in stroke progression and outcome before recanalization
Source: Cell Rep Med. 2026 Mar 31;7(4):102723. doi: 10.1016/j.xcrm.2026.102723 (PMC13130646; doi:10.1016/j.xcrm.2026.102723)
Supplement: Document S2. Article plus supplemental information [file mmc2.pdf]

Cell Reports Medicine

Report

Identifying the role of NLRP3 inflammasome in stroke progression and outcome before recanalization

Graphical abstract

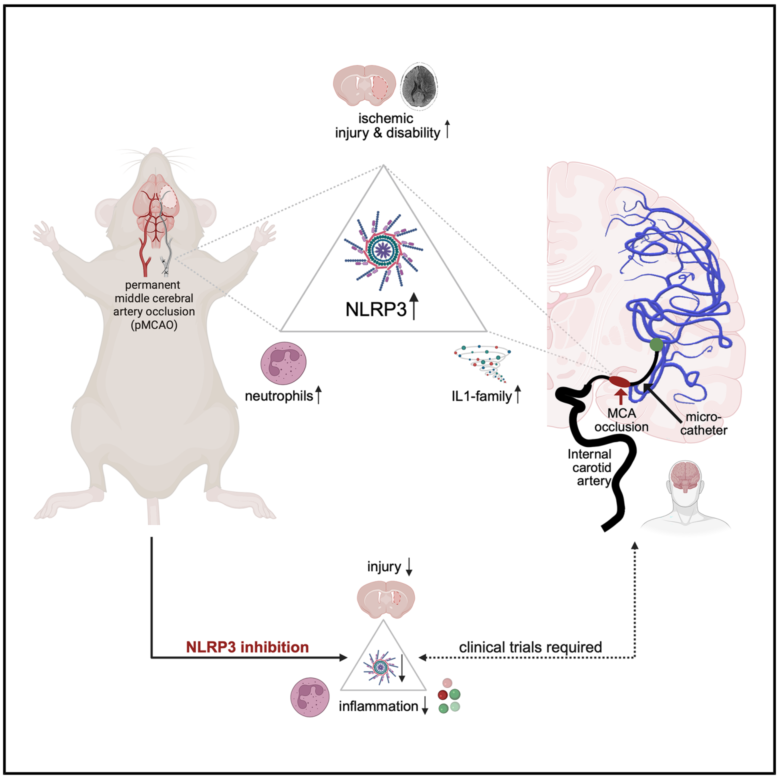

Highlights

- NLRP3 drives hyperacute inflammation during large vessel occlusion in stroke
- NLRP3 inhibition reduces infarct progression already during vessel occlusion
- Cellular NLRP3 levels are elevated in human pial blood before thrombectomy
- NLRP3^{+} cells in human pial blood correlate with infarct size and outcome

Authors

Maximilian Bellut, Alexander M. Kollikowski, Marius L. Vogt, ..., Mirko Pham, Guido Stoll, Michael K. Schuhmann

Correspondence

bellut_m@ukw.de (M.B.), schuhmann_m@ukw.de (M.K.S.)

In brief

Bellut et al. show that the NLRP3 inflammasome drives hyperacute inflammation and infarct progression during large vessel occlusion in stroke. Pharmacological inhibition reduces infarct growth in mice. NLRP3-positive cells in human pial blood before thrombectomy correlate with clinical outcome and highlight a potential therapeutic target prior to recanalization.

Bellut et al., 2026, Cell Reports Medicine 7, 102723
April 21, 2026 © 2026 The Author(s). Published by Elsevier Inc.
https://doi.org/10.1016/j.xcrm.2026.102723

CellPress

Cell Reports Medicine

CellPress
OPEN ACCESS

Report

Identifying the role of NLRP3 inflammasome in stroke progression and outcome before recanalization

Maximilian Bellut,[1,5,*] Alexander M. Kollikowski,[2] Marius L. Vogt,[2] Lukas Rossnagel,[3] Ibrahim Hawwari,[3] Bernardo S. Franklin,[3] Mirko Pham,[2] Guido Stoll,[4] and Michael K. Schuhmann[1,*]
[1]University Hospital Würzburg, Department of Neurology, Würzburg, Germany
[2]University Hospital Würzburg, Department of Neuroradiology, Würzburg, Germany
[3]Institute of Innate Immunity, Medical Faculty, University of Bonn, Bonn, Germany
[4]University Hospital Würzburg, Institute for Experimental Biomedicine, Würzburg, Germany
[5]Lead contact
*Correspondence: bellut_m@ukw.de (M.B.), schuhmann_m@ukw.de (M.K.S.)
https://doi.org/10.1016/j.xcrm.2026.102723

SUMMARY

Acute ischemic stroke (AIS) induces a rapid inflammatory response that partly counteracts the beneficial effects of recanalization by endovascular thrombectomy (EVT). The molecular triggers of inflammation in AIS are still elusive. We analyze the role of the NOD-, LRR-, and pyrin domain-containing protein 3 (NLRP3) inflammasome before recanalization. NLRP3-mRNA levels increase rapidly in the ischemic brain following permanent middle cerebral artery occlusion in mice. NLRP3 protein is primarily expressed by intravascular neutrophils and cerebral endothelium. Blocking NLRP3 activation with the small molecule MCC950 reduces infarct progression and inflammation significantly already during large vessel occlusion. In human AIS, we find similarly increased NLRP3 expression in accumulating leukocytes within pial blood samples taken from the secluded ischemic brain territory immediately before recanalization. The number of NLRP3-positive cells before EVT predicts stroke outcome after 3 months. Our results identify NLRP3 as a promising therapeutic target to attenuate rapid infarct progression prior to recanalization.

INTRODUCTION

Despite significant advances in treating acute ischemic stroke (AIS) caused by large vessel occlusion (LVO) through thrombolysis and/or endovascular thrombectomy (EVT), overall prognosis remains poor. This is largely due to rapid infarct progression during LVO and ischemia/reperfusion (I/R) injury following recanalization.[1] While mechanisms underlying I/R injury have been extensively studied,[2,3] infarct progression before recanalization has been largely neglected. Targeting platelet-driven thromboinflammation by blocking platelet glycoprotein (GP) Ib or GPVI attenuated infarct growth already under LVO in mice.[4,5] In human AIS, platelets locally release chemoattractants and damage-associated molecular patterns (DAMP), resulting in intravascular accumulation of leukocytes.[6,7] The molecular triggers of the inflammation-driven detrimental pathways in AIS are still elusive.

Inflammasomes are critical checkpoint molecules inducing and amplifying innate immune responses with ensuing tissue damage[8–11] after stimulation by pathogen-associated molecular patterns (PAMPs) or DAMPs. In this study, we investigated the functional role of the NLRP3 inflammasome in infarct progression during LVO in experimental stroke. Additionally, we affirmed the relevance of NLRP3 as a predictive marker for stroke outcomes in pial blood samples from AIS patients undergoing EVT.

RESULTS

Study design

To study NLRP3 inflammasome expression and inhibition, we used the permanent middle cerebral artery occlusion (pMCAO) model to induce focal cerebral ischemia in wild-type mice.[12] For gene expression studies, animals were euthanized after 2, 3, or 4 h of pMCAO. Mice treated with MCC950 or vehicle either prophylactically or therapeutically with a delay of 1 or 2 h underwent pMCAO for 4 h and were examined clinically and histologically. Single readouts were validated in a transient MCAO (tMCAO) model with 7 days of reperfusion.[13] Additional readouts in aged mice were performed after tMCAO with 1 day of reperfusion.

In addition, we analyzed pial blood samples collected during EVT under occlusive ischemic conditions by microcatheter aspiration from 18 patients.[14] Recanalization was successful in all studied patients (expanded Thrombolysis In Cerebral Infarction Score (eTICI) $\geq$ 2b50). After recanalization, samples of systemic arterial blood were analogously drawn for direct comparison of NLRP3 expression. We collected radiological findings and clinical data from hospital admission through 3 months post-discharge (basic patient characteristics are summarized in Table 1). Alberta Stroke Program Early CT Score (ASPECTS) and eTICI were

© 2026 The Author(s). Published by Elsevier Inc.
This is an open access article under the CC BY license (http://creativecommons.org/licenses/by/4.0/).

Table 1. Demographic, imaging, treatment, and outcome-related patient characteristics

**Demographics**	
Age, yr, median (IQR)	79 (70–86) (*n* = 18)
Female sex, n (%)	10 (59%) (*n* = 18)
Antiplatelet medication at onset, n (%)	5 (29%) (*n* = 18)
Anticoagulation at onset, n (%)	5 (29%) (*n* = 18)
Patients with previous stroke, n (%)	4 (24%) (*n* = 18)
**Imaging data**	
Baseline ASPECTS, median (IQR)	8 (7–9) (*n* = 18)
**Treatment**	
i.v. rt-PA, n (%)	9 (53%) (*n* = 18)
Intervention: onset-to-recanalization, min, median (IQR)	395 (268–486) (*n* = 18)
**Outcome**	
NIHSS after 72 h, median (IQR)	8 (2–15) (*n* = 16)
mRS after 3 months, median (IQR)	4 (2–5) (*n* = 12)
Recanalization success, eTICI ≥ 2b50, n (%)	18 (100%) (*n* = 18)

Values are presented as number (percentage) for categorical variables and median (IQR) for continuous variables. ASPECTS, Alberta Stroke Program Early CT Score; ICA, internal carotid artery; IQR, interquartile range; i.v. rt-PA, intravenous recombinant tissue plasminogen activator; NIHSS, National Institutes of Health Stroke Scale; mRS, modified Rankin Scale, eTICI, expanded Thrombolysis In Cerebral Infarction score.

determined by finding a consensus between two authors of this study with long-standing experience in stroke imaging, diagnostic cerebral angiography, and vascular neurointervention.

Early NLRP3 upregulation promotes infarct growth under occlusion

Immediately after initiation of MCAO, mRNA levels of NLRP3 and its downstream cytokines interleukin-1β (IL-1b) and IL-18 increased in the ischemic brain hemisphere and remained elevated for several hours (Figure 1A), with only minor changes in the expression of other inflammasomes (Figure S1A). At protein level, NLRP3 immunoreactivity was observed in vascular endothelial cells and intravascular neutrophils but not in neurons (Figure 1B). This contrasts sharply with the reperfusion phase, where NLRP3 predominantly exhibits neuronal expression, as previously demonstrated.[13,15]

Administration of MCC950, a specific and potent NLRP3 inhibitor, significantly reduced infarct progression under LVO not only when given before pMCAO [MCC950 (0h)] but also when applied in a therapeutic setting with a delay of one [MCC950 (1h)] or two [MCC950 (2h)] hours. MCC950 treatment reduced infarct volumes upon histological evaluation [vehicle 67.4 ± 15.3 mm^3; MCC950 (0h): 37.2 ± 10.6 mm^3; MCC950 (1h): 42.9 ± 7.9 mm^3; MCC950 (2h): 49.1 ± 15.8 mm^3; $p < 0.0001$] and significantly improved the neuroscore in mice (Figure 1C). Moreover, neutrophil numbers increased by 800% ($p < 0.0001$) comparing the ischemic with the contralateral hemisphere, while MCC950 treatment reduced the number of ipsilateral neutrophils compared to control mice (decrease of ipsilateral neutrophils in mice treated with MCC950 vs. vehicle by 38%; $p = 0.0083$) (Figure 1D). The NLRP3 activation in immune cells is known to promote the production of the pro-inflammatory cytokines IL-1b and IL-18.[16] IL-1b levels increased in the ischemic hemisphere after stroke but decreased following MCC950 treatment [MCC950 (0h): cortex: −25%, $p = 0.046$; basal ganglia: −23%, $p = 0.0154$] (Figure S1B). Accordingly, pro-IL1b levels increased in the treatment group as a sign of reduced activation, whereas in the vehicle group, they decreased vice versa [AUC vehicle: 137299 vs. MCC 950 (0h): 214646; $p = 0.0013$] (Figure S1C). Together, these findings identify leukocyte, especially neutrophil, and endothelial-cell-derived NLRP3 as a key amplifier of an ultra-early innate immune response.

MCAO-induced caspase-1 and gasdermin D levels are reduced by MCC950

The activation of the IL-1 family depends on active caspase-1, which in turn is directly dependent on inflammasome activation. We demonstrated that cleaved caspase-1 (20 kDa) was significantly less detectable in the treatment groups [MCC950 (0h) −59.9%, $p = 0.0023$] (Figure 2A). We also demonstrated that the pyroptosis marker N-terminal gasdermin D was significantly reduced in the treatment regimens examined (Figure 2B). These effects were paralleled by increased neuronal density and reduced albumin leakage, indicating improved blood-brain barrier integrity (Figures S1D and S1E).

Our investigations in a cohort of mice with a 7-day reperfusion period following tMCAO occlusion demonstrate that this effect is sustained rather than transient (*n* = 6 per group; infarct size in percent of hemisphere: vehicle: 27.85% ± 14.72%; MCC950 13.13% ± 4.25%; $p = 0.0048$).[13] Even after 1 week, significantly reduced levels of activated caspase-1 and N-terminal gasdermin D were observed (Figures 2C and 2D). Importantly, in aged mice (18 months), therapeutic MCC950 likewise attenuated infarct growth under LVO in an I/R-setting, yielding smaller infarcts compared with vehicle, consistent with preserved efficacy in advanced age (*n* = 6 per group; infarct size: vehicle: 71.18 ± 21.27 mm^3; MCC950: 41.02 ± 13.25; $p = 0.014$) (Figures S1F–S1H). Taken together, our data address mechanisms before and shortly after recanalization rather than late functional recovery. While assessments at > 7 days are informative for rehabilitation-dependent outcomes, they interrogate a distinct biological domain from the early NLRP3–IL1 axis that peaks within hours to days after IS.[15]

Leukocyte NLRP3 is higher in ischemic versus systemic blood, and NLRP3-positive cells in pial blood correlate with infarct volume and functional outcome

To translate our murine findings to a clinical context, we examined NLRP3, IL-18, and IL-1b levels in pial blood samples of AIS patients undergoing EVT by staining intracerebral and systemic blood smears and by quantifying plasma IL-18 and IL-1b levels. Quantification of NLRP3-positive cells (ischemic: 24.8 ± 10.3, systemic: 18.1 ± 11.1; $p = 0.0004$) as well as NLRP3 signal intensities per cell showed a significant increase in the pial compared to systemic blood samples (Figure 3A) as well as elevated IL-18 (ischemic: 7.2 ± 5.1 pg/mL,

CellPress
OPEN ACCESS

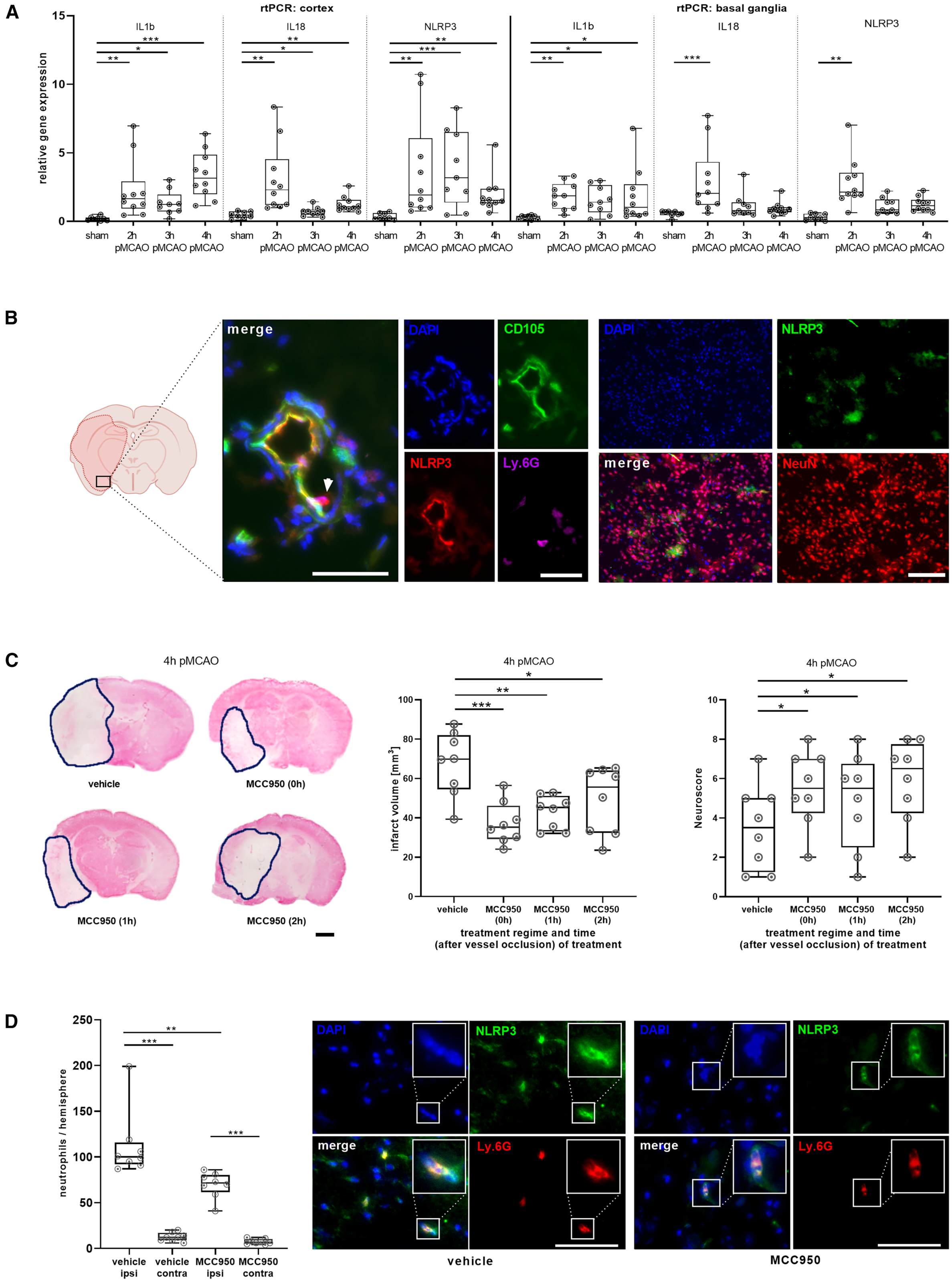

(legend on next page)

systemic: 4.1 ± 1.8 pg/mL; $p = 0.041$) and IL-1b (ischemic: 6.8 ± 4.1 pg/mL, systemic: 4.7 ± 2.2 pg/mL; $p = 0.043$) levels (Figures S2A and S2B, Table S3). Of the 13 cytokines assayed, IL-1b and IL-18 were robustly quantifiable, whereas several additional analytes were below the limit of detection. IL-18 and IL-1b concentrations in the pial blood correlated to cellular NLRP3 expression indicative of NLRP3 activation.[17,18] Notably, neutrophils and leukocytes were significantly elevated in pial compared to systemic samples (Figures S2C), and the number of neutrophils correlated with both pial IL-18 concentration and NLRP3 expression (Figures 3B, S2D, and S2E). Elevated counts of NLRP3-positive leukocytes were associated with more severe strokes, as indicated by an inverse correlation with the Alberta Stroke Program Early CT Score (ASPECTS) before recanalization. Higher NLRP3-positive leukocyte counts also predicted worse outcomes, reflected by higher scores on the National Institutes of Health Stroke Scale (NIHSS) 72 h after EVT and on the modified Rankin Scale (mRS) at discharge as well as at the 3-month follow-up (Figure 3C). Multivariate analysis did not identify any independent predictors, as e.g., age, sex, or prior intravenous (i.v.) lysis therapy, despite NLRP3-positive cells within the ischemic compartment (Tables 1, S1, and S2).

DISCUSSION

Our data provide insights into the regulation of intravascular inflammatory responses in hyperacute stroke before recanalization. We demonstrate a rapid and robust upregulation of the NLRP3 inflammasome in neutrophils and endothelial cells immediately after stroke onset in mice, along with a concurrent increase in the pro-inflammatory cytokines IL-1b and IL-18. It is well established that immune cells, mainly neutrophils, dendritic cells, and macrophages express NLRP3, albeit at low levels under resting conditions, thus requiring upregulation by PAMPs or DAMPs to become functionally active.[16,19] This activation amplifies the inflammatory response and leads to the release of toxic molecules, such as neutrophil extracellular traps and matrix metalloproteinase-9, which damage the blood-brain barrier. This is supported by the successful pharmacological blockade of NLRP3, which mitigated not only infarct progression under LVO but also the inflammatory response. Moreover, the number of NLRP3-positive cells in human pial blood samples obtained within the ischemic brain vasculature predicted both short- and long-term outcomes—even prior to EVT.

Recent studies, particularly in the context of recurrent stroke, have identified cell-free DNA (cfDNA) as a potential DAMP contributing to neuroinflammation.[20] Alongside cfDNA, other DAMPs such as high-mobility group box 1 (HMGB1) have been increasingly recognized for their role in stroke-related immune activation. We previously demonstrated elevated HMGB1 levels in pial blood samples from hyperacute stroke patients with large vessel occlusion, further underscoring the importance of DAMP-mediated signaling in acute cerebrovascular injury.[7] These findings support the broader concept that sterile inflammation in stroke is driven, at least in part, by the release of danger signals. Future investigations into cfDNA in this context could provide valuable insights into the molecular mechanisms of post-stroke inflammation.

The investigation of NLRP3 across multiple studies highlights its relevance in both experimental research and potential clinical translation. Although MCC950 has been evaluated in several models—particularly in transient stroke paradigms as reported by us and others[13,15,21,22]—its role during ongoing vascular occlusion had not previously been addressed. This represents a clinically relevant contribution. Our aim was to closely align experimental conditions with clinical reality. To this end, sampling of pial blood immediately prior to routine thrombectomy captures the pathophysiological environment during large vessel occlusion. This mirrors the conditions modeled by pMCAO in mice.

Importantly, the number of NLRP3-positive cells in human pial blood samples was predictive of long-term clinical outcomes. Consequently, it is essential to consider the additional contribution of I/R injury, which is known to involve NLRP3 inflammasome activation. As previously demonstrated in tMCAO models,[13,15] short occlusion times followed by prompt reperfusion led to significant NLRP3 activation. In these studies, though, stroke outcomes and clinical results significantly improved with MCC950 application after reperfusion. To further substantiate this, we assessed levels of cleaved caspase-1 and N-terminal gasdermin D in the temporary MCAO model, indicating persistent inflammasome activation despite vessel recanalization. These findings support the concept of an NLRP3-driven detrimental inflammatory response in acute stroke commencing

Figure 1. Mouse data: Early NLRP3 upregulation promotes infarct growth under occlusion

(A) Relative gene expression of interleukin-1β (IL-1b), interleukin-18 (IL-18), and NLRP3 in the ischemic cortices and basal ganglia of mice during permanent middle cerebral artery occlusion (pMCAO) for 2, 3, and 4 h or sham treatment; n= 8–10 per group. Data were analyzed by one-way ANOVA.

(B) Representative immunohistochemical multi-channel staining of cerebral vessels within the ischemia (localization graphically shown) of a vehicle-treated mouse after 4 h of pMCAO. Staining for nuclei (DAPI, blue), endothelial cells/vessels (CD105, green), NLRP3 (red), and neutrophils (Ly6G, purple). White arrow indicates an NLRP3-positive intravascular neutrophil. Scale bars = 50 μm (left). Representative immunohistochemical multi-channel staining of the penumbra of a vehicle-treated mouse after 4 h of pMCAO. Staining for nuclei (DAPI, blue), NLRP3 (green) and neurons (NeuN, red). Scale bars = 200 μm (right).

(C) Representative MAP2 stainings of vehicle-, MCC950 (0h)-, MCC950 (1h)-, and MCC950 (2h)-treated mice euthanized after 4 h of pMCAO to visualize infarct volumes. Infarcts circled by a blue line. Scale bars = 2 mm (left). Infarct size comparison in mm^3 of vehicle-, MCC950 (0h)-, MCC950 (1h)-, and MCC950 (2h)-treated mice euthanized after 4 h of pMCAO (middle). Neuroscore as clinical testing of the aforementioned treatment groups for their respective clinical outcomes (right); n= 8 per group. Data were analyzed by one-way ANOVA.

(D) Comparison of the number of hemispherical neutrophils of vehicle- and MCC950 (2h)-treated mice euthanized after 4 h of pMCAO; n = 8 per group (left). Data were analyzed by one-way ANOVA. Representative immunohistochemical multi-channel staining of the penumbra of a vehicle- or MCC950 (2h)-treated mouse after 4 h of pMCAO. Staining for nuclei (DAPI, blue), NLRP3 (green) and neutrophils (Ly6G, red). Scale bars = 100 μm (right). Results are presented as box plots indicating the median, 25th/75th percentile, minimum, and maximum. *$p < 0.05$, **$p < 0.01$, ***$p < 0.001$.

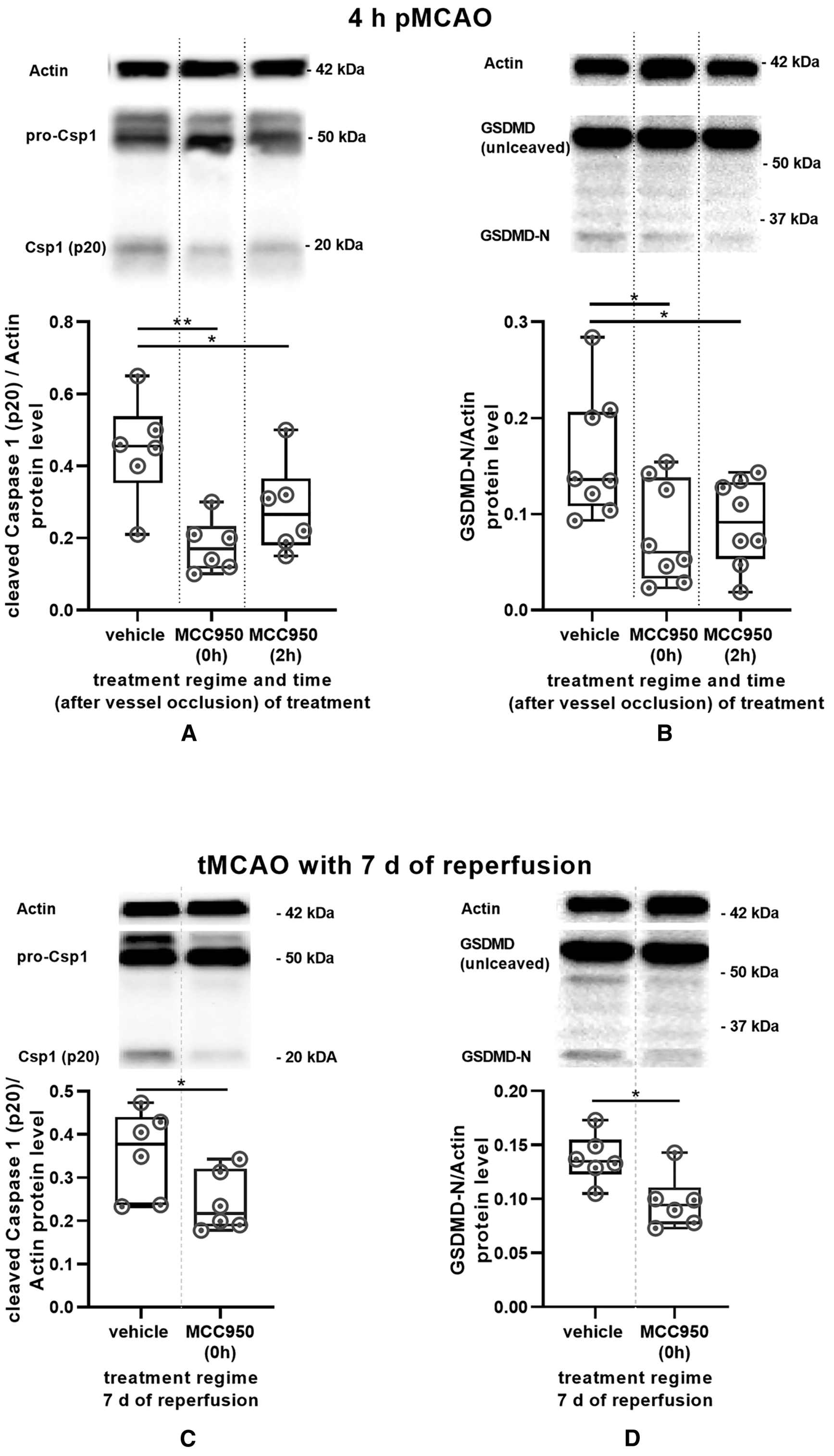

Figure 2. Mouse data: MCAO-induced caspase-1 and gasdermin D are reduced by MCC950

(A) Cleaved caspase-1 (p20) protein content of ipsilateral cortical brain lysates after 4 h of pMCAO of vehicle-, MCC950 (0h), and MCC950 (2h)-treated mice. Actin was used as a loading control for densitometric quantification. Data were analyzed by unpaired *t* test; *n* = 6 per group.

(B) Active gasdermin D (N-terminal) protein content of ipsilateral cortical brain lysates after 4 h of pMCAO of vehicle-, MCC950 (0h), and MCC950 (2h)-treated mice. Actin was used as a loading control for densitometric quantification. Data were analyzed by unpaired *t* test; *n* = 8 per group.

(C) Active caspase-1 (p20) protein content of ipsilateral cortical brain lysates after 7 days of reperfusion following 30 min tMCAO of vehicle- and MCC950 (0h)-treated mice. Actin was used as a loading control for densitometric quantification. Data were analyzed by unpaired *t* test; *n* = 6 per group.

(D) Active gasdermin D (N-terminal) protein content of ipsilateral cortical brain lysates after 7 days of reperfusion following 30 min tMCAO of vehicle- and MCC950 (0h)-treated mice. Actin was used as a loading control for densitometric quantification. Data were analyzed by unpaired *t* test; *n* = 6 per group. Results are presented as box plots indicating the median, 25th/75th percentile, minimum, and maximum. *$p < 0.05$, **$p < 0.01$, ***$p < 0.001$.

during large vessel occlusion but persisting despite successful recanalization.

We were able to demonstrate that targeting NLRP3 offers a promising therapeutic approach to limit stroke progression across both the occlusive and post-occlusive phases of AIS. This is particularly important, as NLRP3 inhibitors are currently in various phase II/III trials for different inflammatory indications such as gout or arthritis and have thus already overcome initial hurdles on the path to approval.[23]

Limitations of the study

This pilot study was conducted in young, healthy male mice, and the efficacy of NLRP3 inhibition was confirmed in aged male mice. Future studies should include comorbid and female cohorts to further investigate the pathophysiological role of NLRP3 and the therapeutic potential of its inhibition in pMCAO and tMCAO models. Female cohorts will require careful experimental planning, including dose-finding and pharmacological characterization, monitoring of the estrous cycle and hormonal status, and sufficiently powered group sizes to account for sex- and cycle-related variability. These considerations were beyond the scope of the present pilot study.

In addition to acute outcome measures, future studies should assess long-term functional recovery using behavioral and

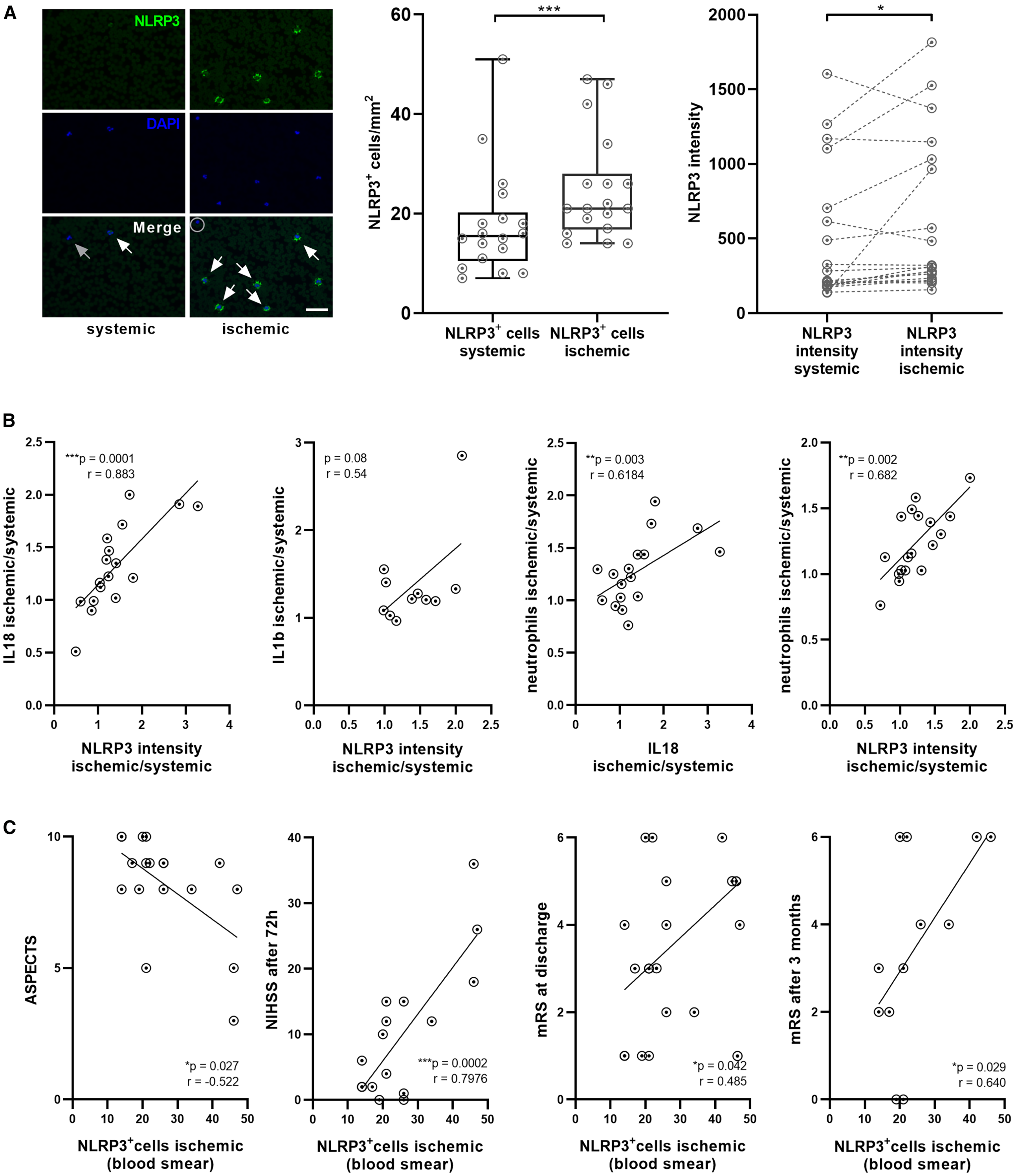

Figure 3. Human data: Leukocyte NLRP3 is higher in ischemic versus systemic blood and NLRP3-positive cells in pial blood correlate with infarct volume and functional outcome

(A) Representative immunofluorescence stainings of systemic and ischemic blood smears stained for NLRP3 (green) and DAPI (blue); white arrows mark NLRP3/DAPI double-positive cells, gray arrows mark NLRP3-negative nucleated cells, circles depict lymphocytes with spherical nuclei in comparison to multilobulated nuclei of neutrophils. 20× objective; scale bars = 50 μm (left). Count of NLRP3-positive cells per mm^2 at corresponding sections of blood smears of systemic or

(legend continued on next page)

CellPress
OPEN ACCESS

cognitive endpoints to better capture clinically relevant effects. To enhance the robustness and translational potential of our findings, replication in independent laboratories or within a multi-center preclinical network is recommended. Similarly, validation of our clinical findings in larger, independent AIS patient cohorts is essential.

RESOURCE AVAILABILITY

Lead contact

Further information and requests for resources should be directed to and will be fulfilled by the lead contact, Maximilian Bellut (bellut_m@ukw.de).

Materials availability

This study did not generate new unique reagents.

Data and code availability

- The data reported in this paper will be shared by the lead contact upon reasonable request.
- This paper does not report original code.
- Any additional information required to reanalyze the data reported in this work is available from the lead contact upon request.

ACKNOWLEDGMENTS

We thank Susanne Hellmig, Gabi Köllner, and Hai Yen Hagn for excellent technical assistance. We thank all neurointerventionalists and radiology technicians of the Department of Neuroradiology for their support in data acquisition and patient handling. Moreover, we thank Salie Maasewerd and Matilde B. Vasconcelos for their continuous scientific advice. This research was funded by Else-Kröner-Fresenius-Stiftung (EKFS) (2023_EKEA.187 to M.B.) and the Interdisciplinary Center for Clinical Research (IZKF) at the University of Würzburg (T-516 to A.M.K. and M.K.S.). M.K.S. was supported by the Hentschel Stiftung, too.

AUTHOR CONTRIBUTIONS

M.B., G.S., and M.K.S. performed study concept and design. M.B., L.R., I.H., and A.M.K. performed experiments. M.B., M.L.V., L.R., A.M.K., G.S., and M.K.S. analyzed data. M.B., A.M.K., B.S.F., G.S., M.P., and M.K.S. discussed results and provided important intellectual content throughout the study. M.B., G.S., and M.K.S. wrote the paper with input and approval from all authors.

DECLARATION OF INTERESTS

The authors declare no competing interests.

STAR★METHODS

Detailed methods are provided in the online version of this paper and include the following:

- KEY RESOURCES TABLE
- EXPERIMENTAL MODEL AND STUDY PARTICIPANT DETAILS
  - Mouse model
  - Human samples
- METHOD DETAILS
  - Animal treatment
  - Ischemia model
  - Cytokine quantification
  - Cell counts and blood smear preparation
  - Protein extraction and Western blot analysis
  - Capillary western blot (Wes) electrophoresis and immunoblotting
  - Quantitative real-time PCR (PCR)
  - Immunohistochemistry
- QUANTIFICATION AND STATISTICAL ANALYSIS
  - Statistical analysis

SUPPLEMENTAL INFORMATION

Supplemental information can be found online at https://doi.org/10.1016/j.xcrm.2026.102723.

Received: November 1, 2024
Revised: October 13, 2025
Accepted: March 9, 2026
Published: March 31, 2026

REFERENCES

1. Wechsler, L.R., Adeoye, O., Alemseged, F., Bahr-Hosseini, M., Deljkich, E., Favilla, C., Fisher, M., Grotta, J., Hill, M.D., Kamel, H., et al. (2023). Most Promising Approaches to Improve Stroke Outcomes: The Stroke Treatment Academic Industry Roundtable XII Workshop. Stroke *54*, 3202–3213. https://doi.org/10.1161/STROKEAHA.123.044279.
2. Bai, J., and Lyden, P.D. (2015). Revisiting Cerebral Postischemic Reperfusion Injury: New Insights in Understanding Reperfusion Failure, Hemorrhage, and Edema. Int. J. Stroke *10*, 143–152. https://doi.org/10.1111/ijs.12434.
3. Stoll, G., and Nieswandt, B. (2019). Thrombo-inflammation in acute ischaemic stroke — implications for treatment. Nat. Rev. Neurol. *15*, 473–481. https://doi.org/10.1038/s41582-019-0221-1.
4. Schuhmann, M.K., Bieber, M., Franke, M., Kollikowski, A.M., Stegner, D., Heinze, K.G., Nieswandt, B., Pham, M., and Stoll, G. (2021). Platelets and lymphocytes drive progressive penumbral tissue loss during middle cerebral artery occlusion in mice. J. Neuroinflammation *18*, 46. https://doi.org/10.1186/s12974-021-02095-1.
5. Bieber, M., Schuhmann, M.K., Kollikowski, A.M., Stegner, D., Nieswandt, B., Pham, M., and Stoll, G. (2021). Targeting platelet glycoprotein VI attenuates progressive ischemic brain damage before recanalization during middle cerebral artery occlusion in mice. Exp. Neurol. *344*, 113804. https://doi.org/10.1016/j.expneurol.2021.113804.
6. Kollikowski, A.M., Pham, M., März, A.G., Papp, L., Nieswandt, B., Stoll, G., and Schuhmann, M.K. (2021). Platelet Activation and Chemokine Release Are Related to Local Neutrophil-Dominant Inflammation During Hyperacute Human Stroke. Transl Stroke Res *13*, 364–369. https://doi.org/10.1007/s12975-021-00938-w.
7. Schuhmann, M.K., Kollikowski, A.M., März, A.G., Bieber, M., Pham, M., and Stoll, G. (2021). Danger-associated molecular patterns are locally released during occlusion in hyper-acute stroke. Brain Behav. Immun. Health *15*, 100270. https://doi.org/10.1016/j.bbih.2021.100270.

ischemic blood samples (middle); $n = 18$ per group. NLRP3 intensity levels as average of the intensities of all NLRP3-positive cells within each blood smear of systemic or ischemic blood samples (right); $n = 18$ per group. Data were analyzed by paired t test.
(B) Proportion of the ischemic/systemic IL-18 concentration ratio to the ischemic/systemic NLRP3 intensity ratio (first from left). Proportion of the ischemic/systemic IL-1b concentration ratio to the ischemic/systemic NLRP3 intensity ratio (second from left). Proportion of the ischemic/systemic neutrophil count ratio to the ischemic/systemic IL-18 concentration ratio (third from left). Proportion of the ischemic/systemic neutrophil count ratio to the ischemic/systemic NLRP3 intensity ratio (right); $n = 11–18$ per group. Data were analyzed by Spearman correlation.
(C) Correlation between ASPECTS at admission ($n = 18$), NIHSS at 72 h ($n = 16$), mRS at discharge ($n = 18$), mRS 3 months after IS ($n = 12$), and NLRP3$^+$ cells in the ischemic blood. Data were analyzed by Spearman correlation. Results are presented as box plots indicating the median, 25th/75th percentile, minimum and maximum. $^*p < 0.05$, $^{**}p < 0.001$, $^{***}p < 0.001$. r = correlation coefficient.

8. Medzhitov, R., and Janeway, C.A. (1997). Innate Immunity: The Virtues of a Nonclonal System of Recognition. Cell *91*, 295–298. https://doi.org/10.1016/S0092-8674(00)80412-2.

9. Hoffman, H.M., and Broderick, L. (2016). The role of the inflammasome in patients with autoinflammatory diseases. J. Allergy Clin. Immunol. *138*, 3–14. https://doi.org/10.1016/j.jaci.2016.05.001.

10. Guo, H., Callaway, J.B., and Ting, J.P.Y. (2015). Inflammasomes: mechanism of action, role in disease, and therapeutics. Nat. Med. *21*, 677–687. https://doi.org/10.1038/nm.3893.

11. Ravichandran, K.A., and Heneka, M.T. (2024). Inflammasomes in neurological disorders — mechanisms and therapeutic potential. Nat. Rev. Neurol. *20*, 67–83. https://doi.org/10.1038/s41582-023-00915-x.

12. Kleinschnitz, C., Stoll, G., Bendszus, M., Schuh, K., Pauer, H.U., Burfeind, P., Renné, C., Gailani, D., Nieswandt, B., and Renné, T. (2006). Targeting coagulation factor XII provides protection from pathological thrombosis in cerebral ischemia without interfering with hemostasis. J. Exp. Med. *203*, 513–518. https://doi.org/10.1084/jem.20052458.

13. Bellut, M., Bieber, M., Kraft, P., Weber, A.N.R., Stoll, G., and Schuhmann, M.K. (2023). Delayed NLRP3 inflammasome inhibition ameliorates subacute stroke progression in mice. J. Neuroinflammation *20*, 4. https://doi.org/10.1186/s12974-022-02674-w.

14. Kollikowski, A.M., Schuhmann, M.K., Nieswandt, B., Müllges, W., Stoll, G., and Pham, M. (2020). Local Leukocyte Invasion during Hyperacute Human Ischemic Stroke. Ann. Neurol. *87*, 466–479. https://doi.org/10.1002/ana.25665.

15. Franke, M., Bieber, M., Kraft, P., Weber, A.N.R., Stoll, G., and Schuhmann, M.K. (2021). The NLRP3 inflammasome drives inflammation in ischemia/reperfusion injury after transient middle cerebral artery occlusion in mice. Brain Behav. Immun. *92*, 223–233. https://doi.org/10.1016/j.bbi.2020.12.009.

16. Baroja-Mazo, A., Martín-Sánchez, F., Gomez, A.I., Martínez, C.M., Amores-Iniesta, J., Compan, V., Barberà-Cremades, M., Yagüe, J., Ruiz-Ortiz, E., Antón, J., et al. (2014). The NLRP3 inflammasome is released as a particulate danger signal that amplifies the inflammatory response. Nat. Immunol. *15*, 738–748. https://doi.org/10.1038/ni.2919.

17. Yang, F., Wang, Z., Wei, X., Han, H., Meng, X., Zhang, Y., Shi, W., Li, F., Xin, T., Pang, Q., et al. (2014). NLRP3 Deficiency Ameliorates Neurovascular Damage in Experimental Ischemic Stroke. J. Cereb. Blood Flow Metab. *34*, 660–667. https://doi.org/10.1038/jcbfm.2013.242.

18. Wong, R., Lénárt, N., Hill, L., Toms, L., Coutts, G., Martinecz, B., Császár, E., Nyiri, G., Papaemmanouil, A., Waisman, A., et al. (2019). Interleukin-1 mediates ischaemic brain injury via distinct actions on endothelial cells and cholinergic neurons. Brain Behav. Immun. *76*, 126–138. https://doi.org/10.1016/j.bbi.2018.11.012.

19. Lamkanfi, M., and Dixit, V.M. (2014). Mechanisms and Functions of Inflammasomes. Cell *157*, 1013–1022. https://doi.org/10.1016/j.cell.2014.04.007.

20. Cao, J., Roth, S., Zhang, S., Kopczak, A., Mami, S., Asare, Y., Georgakis, M.K., Messerer, D., Horn, A., Shemer, R., et al. (2024). DNA-sensing inflammasomes cause recurrent atherosclerotic stroke. Nature *633*, 433–441. https://doi.org/10.1038/s41586-024-07803-4.

21. Bellut, M., Papp, L., Bieber, M., Kraft, P., Stoll, G., and Schuhmann, M.K. (2021). NLPR3 inflammasome inhibition alleviates hypoxic endothelial cell death in vitro and protects blood–brain barrier integrity in murine stroke. Cell Death Dis. *13*, 20. https://doi.org/10.1038/s41419-021-04379-z.

22. Ismael, S., Zhao, L., Nasoohi, S., and Ishrat, T. (2018). Inhibition of the NLRP3-inflammasome as a potential approach for neuroprotection after stroke. Sci. Rep. *8*, 5971. https://doi.org/10.1038/s41598-018-24350-x.

23. Coll, R.C., Schroder, K., and Pelegrín, P. (2022). NLRP3 and pyroptosis blockers for treating inflammatory diseases. Trends Pharmacol. Sci. *43*, 653–668. https://doi.org/10.1016/j.tips.2022.04.003.

24. Kilkenny, C., Browne, W., Cuthill, I.C., Emerson, M., and Altman, D.G.; National Centre for the Replacement, Refinement and Reduction of Amimals in Research (2011). Animal Research: Reporting *in vivo* Experiments—The ARRIVE Guidelines. J. Cereb. Blood Flow Metab. *31*, 991–993. https://doi.org/10.1038/jcbfm.2010.220.

25. Franke, M., Bieber, M., Stoll, G., and Schuhmann, M.K. (2021). Validity and Efficacy of Methods to Define Blood Brain Barrier Integrity in Experimental Ischemic Strokes: A Comparison of Albumin Western Blot, IgG Western Blot and Albumin Immunofluorescence. MPs *4*, 23. https://doi.org/10.3390/mps4010023.

CellPress
OPEN ACCESS

STAR★METHODS

KEY RESOURCES TABLE

REAGENT or RESOURCE	SOURCE	IDENTIFIER
Antibodies		
Anti-NeuN	Merck	RRID:AB_2298772
Anti-Albumin	Abcam	RRID:AB_10888110
Alexa Fluor(R) 594 anti-mouse Ly-6G	BioLegend	RRID:AB_2563207
Anti-CD105	Abcam	Cat# ab221675; RRID: AB_3718119
anti-NLRP3/NALP3 mAb (Cryo-2)	Adipogen Life Sciences	RRID:AB_2490202
Gasdermin D (E9S1X)	Cell Signaling Technology	RRID:AB_2916333
Goat Anti-Mouse Il-1 beta	R and D Systems	RRID:AB_356450
anti-Caspase-1 (p20) (mouse) mAb (Caspase-1)	Adipogen Life Sciences	RRID:AB_2490248
Peroxidase-AffiniPure Donkey Anti-Mouse IgG	Jackson ImmunoResearch Labs	RRID:AB_2340771
Purified Mouse IgG k Isotype Ctrl	BioLegend	RRID:AB_2801452
Anti-MAP2 Polyclonal Antibody	Abcam	RRID:AB_776174
Anti-β-Actin Antibody	Merck	RRID:AB_476744
Alexa Fluor 488 goat anti-mouse IgG	Thermo Fisher Scientific	RRID:AB_2534069
Alexa Fluor 488 donkey anti-rabbit IgG	Thermo Fisher Scientific	RRID:AB_2535792
Alexa Fluor 647 goat anti-rat IgG	Thermo Fisher Scientific	RRID:AB_141778
Alexa Fluor 546 goat anti-rabbit IgG	Thermo Fisher Scientific	RRID:AB_2534093
Alexa Fluor 488 donkey antirat IgG	Thermo Fisher Scientific	RRID:AB_2535794
Chemicals, peptides, and recombinant proteins		
NLRP3-Inhibitor, MCC950	Merck	Cat# 5381200001
Critical commercial assays		
LEGENDplex™ Human Inflammation Panel 1 (13-plex) with V-bottom Plate	BioLegend	Cat# 740809
12-230 kDa Separation Module	BioTechne	Cat# SM-W001
Anti-Goat Detection Module	BioTechne	Cat# DM-006
TaqMan™ reagent	Applied Biosystems	Cat# N8080234
TaqMan™ NLRP3 Assay	Applied Biosystems	Cat# Mm00840904_m1
TaqMan™ Aim2 Assay	Applied Biosystems	Cat# Mm01295719_m1
TaqMan™ Nlrp1a Assay	Applied Biosystems	Cat# Mm03047263_m1
TaqMan™ Nlrp1b Assay	Applied Biosystems	Cat# Mm01241387_m1
TaqMan™ Nlrc4 Assay	Applied Biosystems	Cat# Mm01239561_m1
TaqMan™ Btk Assay	Applied Biosystems	Cat# Mm00442712_m1
TaqMan™ Il1b Assay	Applied Biosystems	Cat# Mm00434228_m1
TaqMan™ Il18 Assay	Applied Biosystems	Cat# Mm00434225_m1
TaqMan™ Gapdh	Applied Biosystems	Cat# 4352339E
Experimental models: Organisms/strains		
C57Bl/6N Wildtype mice	Charles River Laboratories	RRID:MGI:2159965
Software and algorithms		
GraphPad StatMate 2.00	Dotmatics Limited	RRID:SCR_000306
GraphPad Prism 8.0.2	Dotmatics Limited	RRID:SCR_002798
ImageJ Software 1.52a	National Institutes of Health, USA	https://imagej.nih.gov/ij/
Compass for SimpleWestern software (v6.1.0)	ProteinSimple (Bio-Techne)	https://www.bio-techne.com
LAS X software, Version LAS X 3.1	Leica Microsystems	https://www.leica-microsystems.com
Other		
Silicon rubber–coated 6.0 nylon monofilament	Doccol	Cat# 6023910PK10
DAPI stain	Thermo Fisher Scientific	Cat# P36931

EXPERIMENTAL MODEL AND STUDY PARTICIPANT DETAILS

Mouse model

Animals

All animal experiments were approved by local governmental authorities (Regierung von Unterfranken) and conducted in accordance with the US National Institutes of Health Guidelines for the Care and Use of Laboratory Animals. Moreover, the experiments were designed, performed, and reported according to the Animal Research Reporting of *In Vivo* Experiments (ARRIVE) guidelines.[24] We used 8-10-week old male C57Bl/6 wildtype mice, purchased from Charles River Laboratories (Sulzfeld, Germany), throughout this study. After randomization pMCAO was conducted for either 120, 180 or 240 min. In the case of aged mice, we used 18-month-old C57BL/6 mice from Charles River, which were subjected to a 60-min tMCAO followed by a 23-h reperfusion period. Surgery and evaluation of all readout parameters were performed blinded to the experimental groups. Animals were assigned to the different treatment groups randomly. Mice were maintained under specific pathogen–free conditions in individually ventilated cages (IVC) on a 12-h light/12-h dark cycle at controlled room temperature (21°C–23°C) and relative humidity (45–65%), with *ad libitum* access to standard chow and water. Animals were group-housed (4–5 per cage) and provided with nesting material as environmental enrichment.

Sample size calculation

Assuming a reduction of infarct volume of 30% as functionally relevant and a standard deviation of 20% to the respective mean values, a group size of ≥8 was necessary to show this effect with a power of 0.8 and a probability of a type I error of <0.5 (calculated with GraphPad StatMate 2.00).

Human samples

Patients' eligibility criteria

We analyzed blood samples that were retrieved during MT of 18 white German patients as previously described.[14] The study protocol was approved by the local ethics committee and written informed consent was provided by all participants. Inclusion criteria for clinical and imaging aspects were specified as follows: eligibility required the occurrence of a IS with an initial neurological deficit corresponding to a National Institute of Health Stroke Scale (NIHSS) score of ≥ 6. Before the MT patients got multimodal imaging, including sequential cranial non-contrast computed tomography (CT; Somatom Definition AS; Siemens Healthcare, Erlangen, Germany), CT angiography and (complementary) CT perfusion scans in the given order to rule out hemorrhage or extensive infarction, equivalent to an Alberta Stroke Program Early CT Score (ASPECTS) < 5, to non-invasively determine the occlusion site and to confirm patient eligibility within the extended therapeutic time window of ≤ 24 h, according to current guidelines.

A total of 18 patients (10 female, 8 male) were included. Patients were not allocated to different experimental groups. Instead, a paired within-subject design was applied, in which each patient served as their own control. Ischemic pial blood samples were obtained prior to recanalization, and systemic arterial samples were collected from the same individual immediately after successful reperfusion for direct comparison.

Sampling

EVT procedures have been described in detail before.[14] Immediately after embolus penetration, pial whole blood samples (WBS) were obtained under occlusive ischemic conditions by microcatheter aspiration. A sample of 1 mL of ischemic blood was drawn for laboratory analyses. After recanalization, samples of systemic arterial blood under physiological antegrade flow were analogously drawn by aspiration in the cervical internal carotid artery (ICA) using the same microcatheter as before.

Patient characteristics

The patients' baseline characteristics are presented in Table 1. We collected clinical and radiological characteristics, including ASPECTS at presentation, NIHSS after 72 h, the modified Rankin Scale (mRS) at discharge and 3 months. Recanalization success was determined by the expanded Thrombolysis in Cerebral Infarction (eTICI) scale, with success defined as eTICI ≥ 2b50.

METHOD DETAILS

Animal treatment

Concerning only those experiments with medical treatment. Animals were firstly treated in a prophylactic setting (MCC950 0h), and later in a therapeutic setting after 1 h (MCC950 1h) or 2 h (MCC950 2h) of persisting vessel occlusion with the NLRP3-specific inflammasome inhibitor MCC950 (50 mg/kg; PBS; Merck, Germany). MCC950 was administered by an intraperitoneal injection either directly before occluding the MCA in the prophylactic setting or after either 60 or 120 min of pMCAO in the therapeutic setting.

Ischemia model

We used the pMCAO model to induce focal cerebral ischemia. The experiments were carried out blinded. An independent researcher who was not involved in data analysis coded and randomized the mice. To reduce the variability of our outcome parameters caused by sex-differences only male mice were used in the study. Before pMCAO, the mice were anesthetized with a 2% isoflurane/O_2 mixture. 200 mg/kg body weight metamizol was injected subcutaneously and 4% lidocaine gel applied on the wound margins as analgesia. With a servo-controlled heating blanket, a body core temperature close to 37°C was maintained throughout surgery. After

CellPress
OPEN ACCESS

a midline neck incision, and occlusion of the right proximal common carotid artery and the right external carotid artery a standardized silicon rubber–coated 6.0 nylon monofilament (6023910PK10; Doccol, Sharon, MA, USA) was inserted into the distal right common carotid artery and advanced via the internal carotid artery to occlude the origin of the MCA. Operation time did not exceed 15 min. The same procedure was performed with sham-treated animals except for the occlusion of the MCA. After 120, 180 or 240 min, the mice were re-anesthetized for decapitation. All mice that underwent a medical treatment with MCC950 and their vehicle counterparts had a permanent MCA occlusion for 240 min, while the untreated mice for the gene expression studies had occlusion times of 120 min, 180 min or 240 min. In case of the 7 days reperfusion experiments with the tMCAO model the following setups were chosen: The operation was performed accordingly, after 30 min, the mice were re-anesthetized and the occluding filament removed to allow reperfusion. To reduce infarct variability all mice were operated by the same operator. Operation time did not exceed 15 min. The observation period accounted for 7 days, the mice were re-anesthetized for decapitation. In case of the I/R injury experiments in aged mice the following setups were chosen: The operation was performed accordingly, after 60 min, the mice were re-anesthetized and the occluding filament removed to allow reperfusion. To reduce infarct variability all mice were operated by the same operator. Operation time did not exceed 15 min. The observation period accounted for 1 day, the mice were re-anesthetized for decapitation. Edema-corrected stroke volumes were assessed at the end of the experimental period, based on MAP2- and TTC-stainings.

Cytokine quantification

We quantified the concentrations of IL1β, IFNα2, IFNγ, TNFα, MCP1, IL6, CXCL8 (IL8), IL10, IL12p70, IL17A, IL18, IL23, IL33 by a fluorescent bead immunoassay according to the manufacturer's instructions (740809; BioLegend, San Diego, CA, USA). The 13-cytokine panel (LEGENDplex) that was deliberately selected for high sensitivity to IL1b and IL18. Per-analyte LoD/LoQ are provided in Table S3; values < LoD were treated as left-censored and reported as "< LoD", which does not affect the interpretation of the IL1–focused primary endpoints.

Cell counts and blood smear preparation

We used CPDA-anticoagulated whole blood samples of the different arterial regions for cell counting and the preparation of blood smears. Cell counting was performed after red blood cell lysis and white blood cell (WBC) staining with Tuerk solution (Merck, Darmstadt, Germany) using a Fuchs-Rosenthal counting chamber. To prepare blood smears, a blood droplet (5μL) from whole blood samples was placed on glass slides (R. Langenbrinck, Emmendingen, Germany) and gently spread using the edge of a second glass slide. For histological staining, blood smears were air dried and stained by standard Pappenheim stain (Merck). The numbers of blood leukocyte and neutrophil granulocyte counts were calculated by multiplying the percentages in Pappenheim-stained blood smears with the respective WBC counts.

Protein extraction and Western blot analysis

Dissected cortices and basal ganglia from the mouse brains were homogenized with RIPA buffer (25 mM Tris pH 7.4, 150 mM NaCl, 1% NP-40, 0.1% SDS) containing 4% proteinase inhibitor (cOmpleteTM protease inhibitor cocktail, Thermo Fisher Scientific) and sonified for 10 s. After centrifugation at 15,000 g for 30 min at 4°C supernatants were used for bicinchoninic acid protein assay and subsequent Western Blot analysis. The lysates were mixed with 2 × SDS–PAGE loading buffer (final concentration: 60 mM Tris pH 6.8, 10% beta-mercaptoethanol, 5% SDS, 10% glycerol) at 95°C for 10 min. 20 μg of total protein was loaded on the gel, electrophoresed and transferred to a nitrocellulose membrane. After blocking for 30 min with blocking buffer (5% nonfat dry milk, PBS, 0.05% Tween 20) membranes were incubated with the primary antibody anti-Caspase 1 (1:100). As secondary antibodies Peroxidase AffiniPure Donkey Anti-Mouse IgG (1:2000) were used.

Capillary western blot (Wes) electrophoresis and immunoblotting

WES was conducted using the ProteinSimple WES system. Whole tissue extracts were diluted in 0.1x sample buffer prior to analysis. Four parts of the diluted sample were mixed with one part of 5x fluorescent master mix, which contained 5x sample buffer, 5x fluorescent standard, and 200 mM DTT. The mixture was then heated at 95°C for 5 min to denature the proteins. The fluorescent master mix included three fluorescent proteins that served as internal markers to normalize the distance between capillaries, as the molecular weight ladder was applied only to the first capillary and each capillary operates independently. Following denaturation, the prepared samples, blocking reagent, primary antibodies (diluted 1:50 for mouse Caspase 1 and mouse pro-IL1b), HRP-conjugated secondary antibodies, and chemiluminescent substrate were loaded into the designated wells of a test plate. A biotinylated molecular weight ladder was used as a reference for molecular weight standards.

Once the plate was loaded, separation electrophoresis and immunodetection were performed using the fully automated WES capillary system. The Chemiluminescence intensity is assessed through electrophoretograms generated by Compass for SimpleWestern software v6.1.0. The areas under the curve (AUC) were detected to present for the relative amount of special proteins. A linear regression fit was used to determine the linear dynamic ranges of special proteins.

Quantitative real-time PCR (PCR)

For quantitative real-time PCR analysis focal cerebral ischemia was induced and animals were scarified 2, 3 and 4 h after stroke induction and analysis was performed according to standard procedures. First we separated the cortices and basal ganglia tissue from

both hemispheres for RNA isolation. After homogenization with TRIzol Reagent (1 mL per 100 mg tissue), chloroform was added and samples were centrifuged at 12.000 g for 5 min at 4°C. The upper aqueous phase was collected and mixed with isopropyl alcohol for RNA precipitation, washed, dissolved in TE buffer and finally quantified spectrophotometrically. 1 μg of total RNA were used for reverse transcription with the TaqMan Reverse Transcription Reagents according to the manufactureŕs protocol using random hexamers. We used primers for IL1b, IL18, NLRP3, AIM2, NLRC4, NLRP1a, NLRP1b and BTK. GAPDH was used as endogenous control. PCR was performed with equal amounts of cyclic deoxyribonucleic acid and water control in the StepOnePlusTM Real-Time PCR System (Applied Biosystems) using the TaqMan Universal 2X PCR Master Mix (Applied Biosystems). Each sample was measured in duplicate and all data points examined for integrity by analysis of the amplification plot. The comparative cycle threshold method was used for relative quantification of gene expression as described elsewhere.

Immunohistochemistry

Murine brain tissue was cut in 2-mm-thick coronal sections, embedded in Tissue-Tek OCT compound and frozen. Brain sections were cut on a cryostat into 10-μm thin slices and used for all analysis. For immunohistochemistry, slices were post-fixated with ice-cold 100% methanol. Immunohistochemistry was performed according to standard procedures.[25] Mouse brains were stained for DAPI (#P36931, Thermo Fisher Scientific, Waltham, MA, USA), anti-NeuN (MAB377, 1:100, Merck-Millipore, Burlington, MA, USA), anti-albumin (ab106582, 1:200, Abcam, Cambridge, UK), anti-Ly6G (127636, 1:50, BioLegend, San Diego, CA, USA), anti-CD105 (ab 221675, 1:50, Abcam, Cambridge, UK) and anti-NLRP3 (AG-20B-0014-C100, 1:100, Adipogen Life Sciences, San Diego, CA, USA). Secondary antibodies were used in a dilution of 1:100. For measurement of the NeuN/albumin intensity, images at the level of the basal ganglia (0.4, 0.45 and 0.5 mm anterior from bregma) were recorded with the microscope (Leica DMi8 equipped with the DMC 2900/DFC 3000G camera control and LAS X software; Leica, Wetzlar, Germany). Subsequently, after converting the images into 16-bit black and white files, the intensity of the NeuN/albumin stainings was compared between the ipsilesional and contralesional hemispheres with ImageJ. For this purpose, the ratio of the overall intensity scores of the ipsilesional and contralesional hemisphere was calculated and served as a comparison parameter among each single animal.

Whole blood smears were stained with anti-NLRP3 (AG-20B-0014-C100, 1:100, Adipogen Life Sciences, San Diego, CA, USA) and DAPI (#P36931, Thermo Fisher Scientific, Waltham, MA, USA). Images were recorded with a Leica DMi8 microscope (Leica DMi8 equipped with the DMC 2900/DFC 3000G camera control and LAS X software; Leica, Wetzlar, Germany). Intensities of NLRP3 were quantified as described in[25] and compared between the single BS with ImageJ (ImageJ Analysis Software 1.52a; National Institutes of Health, Bethesda, MD, USA). The number of NLRP3-positive cells was counted in respective areas of 1 mm^2 with equally distributed erythrocytes in the background to ensure uniform analysis.

QUANTIFICATION AND STATISTICAL ANALYSIS

Statistical analysis

Results are presented as boxplots indicating the median, $25^{th}/75^{th}$ percentile, minimum and maximum. Error bars show the standard error of the mean. For statistical analysis, the GraphPad Prism 8 software was used. Data were tested for Gaussian distribution with the D'Agostino-Pearson omnibus normality test and analyzed by two-tailed t-tests. In the absence of normal distribution, we employed the Kruskal-Wallis test. In the case of comparisons involving more than two groups we applied 1-way analysis of variance (ANOVA) with post hoc Bonferroni adjustment for p values. To assess correlations, we used Spearman correlation. We performed an exploratory univariate regression analysis of patient characteristics, NLRP3+ cells and their association with early infarct volume and outcome measures as well as a best-fit prediction model with multivariable regression using stepwise-backwards selection to investigate potential associations between the dependent variables and independent patient characteristics. Probability values <0.05 were considered to indicate statistically significant results. p-values were indicated as follows: $^{*}p < 0.05$, $^{**}p < 0.01$, $^{***}p < 0.001$.

Cell Reports Medicine, Volume 7

Supplemental information

Identifying the role of NLRP3 inflammasome

in stroke progression and outcome

before recanalization

Maximilian Bellut, Alexander M. Kollikowski, Marius L. Vogt, Lukas Rossnagel, Ibrahim Hawwari, Bernardo S. Franklin, Mirko Pham, Guido Stoll, and Michael K. Schuhmann

Supplemental Figure 1 (related to Figure 1): Hyperacute inflammasome activation and neuroprotective effects of MCC950 during experimental cerebral ischemia

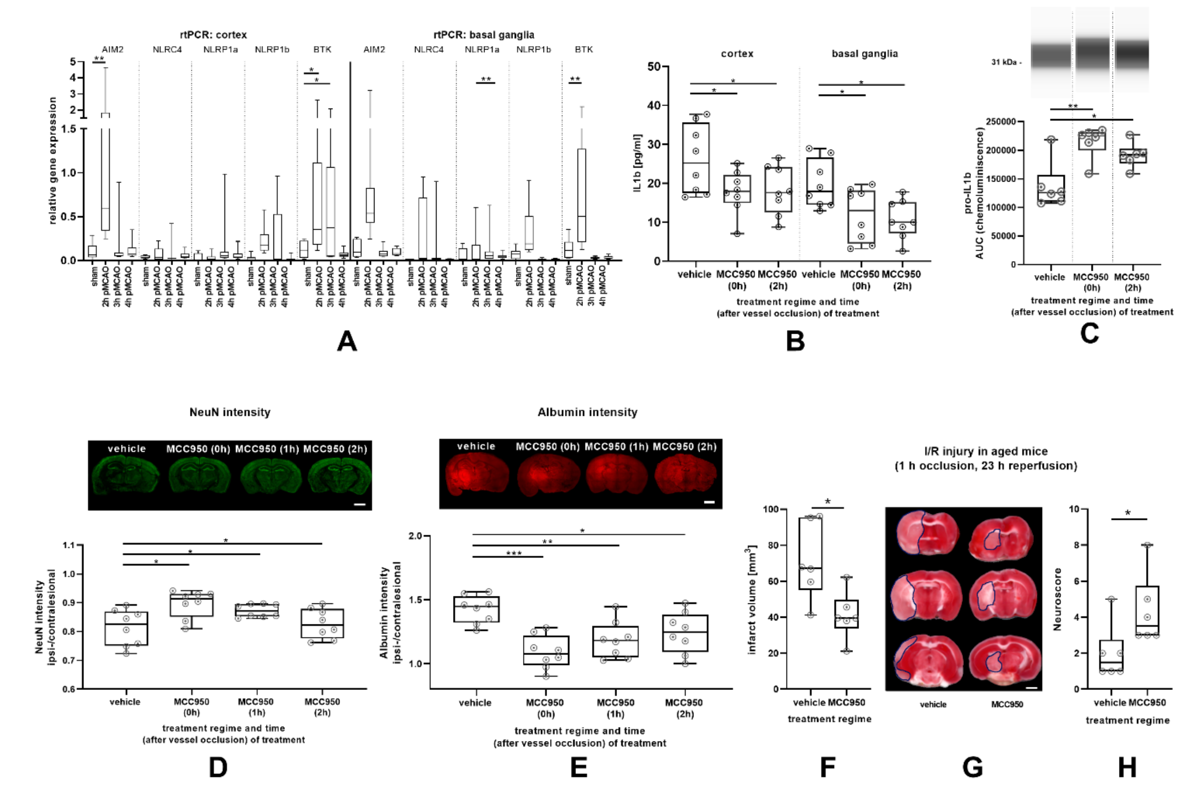

Suppl. Fig. 1 (A) Relative gene expression of AIM2, NLRC4, NLRP1a, NLRP1b and BTK in the ischemic cortices and basal ganglia of mice during permanent middle cerebral artery occlusion (pMCAO) for 2 h, 3 h and 4 h or sham-treatment; n = 8-10 per group. Data was analyzed by 1-way ANOVA. (B) Cytometric bead array to quantify IL1b protein levels of ipsilateral cortical and basal ganglial brain lysates after 4 h of MCAO of vehicle-, MCC950 (0h)- and MCC950 (2h)-treated mice; n = 8 per group. Data was analyzed by 1-way ANOVA. (C) WES capillary electrophoresis and immunoblotting of pro-IL1b in ipsilateral cortical brain lysates after 4 h of MCAO of vehicle-, MCC950 (0h)- and MCC950 (2h)-treated mice. Top: Representative immunoblotting results of 1 individual per group as assigned. Bottom: pro-IL1b levels measured by WES capillary electrophoresis quantified as area under the curve (AUC). Data was analyzed by unpaired t-test; n = 6 per group. (D) Top: Representative immunohistochemical stainings of NeuN (neurons, green) of coronary brain slices after 4 h of MCAO of vehicle-, MCC950 (0h), MCC950 (1h)- and MCC950 (2h)-treated mice using 5 × objective. Scale bar = 2 mm. Bottom: Ratio of ipsilesional to contralesional NeuN intensity of the aforementioned groups; n = 8 per group. Data was analyzed by 1-way ANOVA. (E) Top: Representative immunohistochemical stainings of Albumin (red) of coronary brain slices after 4h of MCAO of vehicle-, MCC950 (0h), MCC950 (1h)- and MCC950 (2h)-treated mice using 5 × objective. Scale bar = 2 mm. Bottom: Ratio of ipsilesional to contralesional Albumin intensity of the aforementioned groups; n = 8 per group. Data was analyzed by 1-way ANOVA. (F) Infarct size comparison in mm^3 of vehicle- and MCC950-treated mice euthanized after 1h of tMCAO and 23 h of reperfusion; n = 6 per group. (G) Representative TTC stainings of vehicle- and MCC950-treated mice euthanized after 1h of tMCAO and 23h of reperfusion to visualize infarct volumes. Infarcts circled by a blue line. Scale bar = 2 mm. (H) Neuroscore as clinical testing of the aforementioned treatment groups for their respective clinical outcomes; n = 6 per group. Data were analyzed by 1-way ANOVA. Results are presented as box plots indicating the median, $25^{th}/75^{th}$ percentile, minimum and maximum. *$p < 0.05$, **$p < 0.01$, ***$p < 0.001$.

Supplemental Figure 2 (related to Figure 3): Compartment-specific inflammasome signaling and immune cell dynamics in systemic versus ischemic human stroke blood

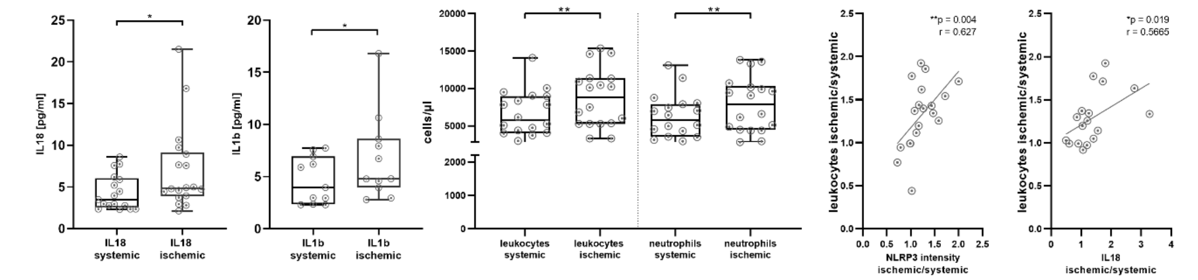

Suppl. Fig. 2. (A) Cytometric bead array to detect IL18 concentrations in the systemic and ischemic compartment from human blood samples. Data was analyzed by paired t-test; n = 18 (B) Cytometric bead array to detect IL1b concentrations in the systemic and ischemic compartment from human blood samples. Data was analyzed by paired t-test; n = 11. (C) Total leukocyte and neutrophil counts of systemic arterial blood versus local occlusive blood. Data was analyzed by unpaired t-test; n = 18. (D) Proportion of the ischemic/systemic leukocyte count ratio to the ischemic/systemic NLRP3 intensity ratio. Data was analyzed by Spearman correlation; n = 18. (E) Proportion of the ischemic/systemic leukocyte count ratio to the ischemic/systemic IL18 concentration ratio. Data was analyzed by Spearman correlation; n = 18. Results are presented as box plots indicating the median, $25^{th}/75^{th}$ percentile, minimum and maximum. *p < 0.05, **p < 0.01, ***p < 0.001.

**Baseline ASPECTS**						
**Predictor variable**	**B**	**SE**	**95% CI**	**t**	R^2	**p** value
NLRP3$^+$ cells$_{ischemic}$	-0.097	0.035	-0.17 – 0.023	-2.80	0.33	0.013
Age	0.033	0.035	-0.042 – 0.11	0.93	0.051	0.37
Female sex	0.3	0.95	-1.71 – 2.31	0.32	0.0062	0.76
History of prior stroke	1.07	1.11	-1.27 – 3.42	0.97	0.055	0.35
Unknown onset	0.6	1.05	-1.62 – 2.82	0.57	0.02	0.57
Antiplatelet drugs	0.6	1.05	-1.62 – 2.82	0.57	0.02	0.57
Anticoagulants	1.15	1.02	-1.0009 – 3.31	1.14	0.074	0.27
**NIHSS after 72h**						
**Predictor variable**	**B**	**SE**	**95% CI**	**t**	R^2	**p** value
NLRP3$^+$ cells$_{ischemic}$	0.71	0.14	0.4 – 1.022	4.9	0.63	0.0002
Age	0.042	0.2	-0.39 – 0.47	0.21	0.003	0.84
Female sex	-6.19	5.098	-17.12 – 4.74	-1.21	0.095	0.24
History of prior stroke	-8.26	6.45	-22.08 – 5.57	-1.28	0.1	0.22
Unknown onset	-3.16	5.67	-15.33 – 9.0059	-0.56	0.022	0.59
Onset-to-recanalization time	-0.0058	0.014	-0.037 – 0.025	-0.41	0.019	0.69
Antiplatelet drugs	-6.5	5.89	-19.13 – 6.13	-1.1	0.08	0.29
Anticoagulants	1.15	1.016	-1.0009 – 3.31	1.13	0.075	0.27
Intravenous alteplase treatment	7.71	4.95	-2.89 – 18.33	1.56	0.15	0.14
**mRS at discharge**						
**Predictor variable**	**B**	**SE**	**95% CI**	**t**	R^2	**p** value
NLRP3$^+$ cells$_{ischemic}$	0.075	0.034	0.00039 – 0.15	2.13	0.22	0.049
Age	0.037	0.033	-0.032 – 0.11	1.14	0.076	0.27
Female sex	0.35	0.89	-1.53 – 2.23	0.39	0.0096	0.69
History of prior stroke	-0.89	1.041	-3.1 – 1.31	-0.86	0.044	0.4
Unknown onset	-0.89	0.96	-2.93 – 1.15	-0.93	0.051	0.37
Onset-to-recanalization time	0.0019	0.0017	-0,0018 - 0.0057	1.13	0.1	0.28
Antiplatelet drugs	0.49	0.98	-1.59 – 2.57	0.5	0.016	0.62
Anticoagulants	-0.62	0.98	-2,69 – 1.45	-0.63	0.024	0.54
Intravenous alteplase treatment	1.33	0.82	-0.41 – 3.072	1.63	0.14	0.12
**mRS after 3 months**						
**Predictor variable**	**B**	**SE**	**95% CI**	**t**	R^2	**p** value
NLRP3$^+$ cells$_{ischemic}$	0.12	0.055	0.0023 – 0.24	2.27	0.34	0.047
Age	0.011	0.047	-0.094 – 0.12	0.23	0.0052	0.82
Female sex	-0.86	1.35	-3.86 – 2.14	-0.64	0.039	0.54
History of prior stroke	0.6	1.81	-3.43 – 4.63	0.33	0.011	0.75
Unknown onset	-0.89	0.96	-2.93 – 1.15	-0.93	0.051	0.368
Onset-to-recanalization time	0.0061	0.0068	-0.0096 – 0.022	0.89	0.091	0.39
Antiplatelet drugs	-0.22	1.56	-3.7 – 3.26	-0.14	0.002	0.89
Anticoagulants	0.38	1.43	-2.81 – 3.56	0.26	0.0068	0.79
Intravenous alteplase treatment	0.86	1.35	-2.14 – 3.86	0.64	0.039	0.54

Suppl. Table 1: Exploratory univariate regression analysis of patient characteristics, NLRP3$^+$ cells and their association with early infarct volume and outcome measures. ASPECTS, Alberta Stroke Program Early CT Score; B, Coefficient; CI, Confidence interval; mRS, modified Rankin Scale; NIHSS, National Institutes of Health Stroke Scale; R2, Coefficient of determination; SE, standard error.

**mRS after 3 months**						
**Predictor variable**	**B**	**SE**	**95% CI**	**t**	**R^2**	**p** value
Constant	-1.24	1.29	-4.4 – 1.91	-0.96		0.37
NLRP3$^+$ cells$_{ischemic}$	0.16	0.048	0.038 – 0.27	3.22	0.63	0.018

Suppl. Table 2: Best-fit model in prediction of long-term outcome at 3-month follow-up. Multivariable regression using stepwise-backwards selection (cut-off point of p > 0.1); Covariates: age, NIHSS (National Institutes of Health Stroke Scale), onset-to-recanalization time; R^2-adjusted = 0.57; multiple correlation coefficient = 0.79. B, Coefficient; CI, Confidence interval; R^2, Coefficient of determination; SE, standard error.

**Cytokines**	***IL18***	***IFNγ***	***IL10***	***IL8***	***IL1b***	***IL6***	***MCP1***	***IL12p70***	***IL23***	***IFNα2***	***IL17A***	***TNFα***	***IL33***
**Pat. no. below detection limit**	0	8	1	1	7	2	0	17	16	18	18	18	18
**mean conc. systemic**	4.35 (±2.12)	2.69 (±2.35)	9.04 (±8.25)	255.17 (±210.8)	4.65 (±2.23)	26.25 (±22.10)	285.14 (±149.6)	4.53 -	11.92 (±1.76)	< LoD	< LoD	< LoD	< LoD
**mean conc. ischemic**	7.05 (±2.09)	3.89 (±3.15)	10.76 (±8.91)	434.68 (±250.7)	6.78 (±4.14)	37.65 (±26.47)	294.98 (±155.1)	7.43 -	6.53 (±0.69)	< LoD	< LoD	< LoD	< LoD
**p-value cytokine conc. i/s**	***0.048***	0.051	0.453	0.156	***0.043***	***0.023***	0.811	-	-	-	-	-	-
**p-value correlation cytokine conc. [i/s]/ NLRP3 intensity [i/s]**	***0.0001***	0.711	0.128	0.413	*0.08*	0.217	0.685	-	-	-	-	-	-
**r^2 correlation cytokine conc. [i/s]/ NLRP3 intensity [i/s]**	0.88	0.02	0.14	0.04	0.39	0.11	0.11	-	-	-	-	-	-

Suppl. Table 3: Panel showing cytokine concentrations (IL18, IFNγ, IL10, IL8, IL1b, IL6, MCP1, IL12p70, IL23) in systemic and ischemic (pial) blood samples from stroke patients undergoing endovascular thrombectomy (n = 18). The panel was selected for IL1b and IL18 detection; several other analytes were below the limit of detection, reported as < LoD
For each cytokine, the following information is provided: number of patients with levels below the detection limit, mean concentration in systemic and ischemic blood samples, p-value comparing systemic vs. ischemic concentrations and the correlation (p-value and r^2) between the ischemic/systemic cytokine ratio and the corresponding ischemic/systemic NLRP3 intensity ratio. i/s = ischemic/systemic.
